# X-radiation enhances the collagen type I strap formation and migration potentials of colon cancer cells

**DOI:** 10.18632/oncotarget.12111

**Published:** 2016-09-19

**Authors:** Stephanie Blockhuys, Na Liu, Nisha Rani Agarwal, Annika Enejder, Vesa Loitto, Xiao-Feng Sun

**Affiliations:** ^1^ Department of Oncology and Department of Clinical and Experimental Medicine, Linköping University, Linköping, Sweden; ^2^ Medical Microbiology, Department of Clinical and Experimental Medicine, Linköping University, Linköping, Sweden; ^3^ Molecular Microscopy, Department of Biology and Biological Engineering, Chalmers University of Technology, Gothenburg, Sweden

**Keywords:** collagen type 1, colorectal cancer, X-radiation, cell migration, integrin β1

## Abstract

Rectal cancer treatment still fails with local and distant relapses of the disease. It is hypothesized that radiotherapy could stimulate cancer cell dissemination and metastasis. In this study, we evaluated the effect of X-radiation on collagen type I strap formation potential, i.e. matrix remodeling associated with mesenchymal cell migration, and behaviors of SW480, SW620, HCT116 p53^+/+^ and HCT116 p53^−/−^ colon cancer cells. We determined a radiation-induced increase in collagen type I strap formation and migration potentials of SW480 and HCT116 p53^+/+^. Further studies with HCT116 p53^+/+^, indicated that after X-radiation strap forming cells have an increased motility. More, we detected a decrease in adhesion potential and mature integrin β1 expression, but no change in non-muscle myosin II expression for HCT116 p53^+/+^ after X-radiation. Integrin β1 neutralization resulted in a decreased cell adhesion and collagen type I strap formation in both sham and X-radiated conditions. Our study indicates collagen type I strap formation as a potential mechanism of colon cancer cells with increased migration potential after X-radiation, and suggests that other molecules than integrin β1 and non-muscle myosin II are responsible for the radiation-induced collagen type I strap formation potential of colon cancer cells. This work encourages further molecular investigation of radiation-induced migration to improve rectal cancer treatment outcome.

## INTRODUCTION

Surgery is the mainstay of curative rectal cancer treatments, and radiotherapy in combination with chemotherapy significantly decreases local relapse and improves overall survival [1]. However, treatment failure with local and distant recurrence of the disease is still a major clinical problem [2, 3]. Besides the therapeutic effect, radiation may as well stimulate cancer metastasis via various molecular mechanisms [4, 5]. Cancer cell migration is a key process of metastasis [6] and the mechanistic elucidation of radiation-induced (RI) migration is crucial to improve rectal cancer treatment outcome.

During cancer metastasis, cancer cells migrate through a heterogeneous tumor microenvironment according a mesenchymal or amoeboid pattern. Mesenchymal migrating cancer cells actively remodel their surrounding matrix by deposition of new extracellular matrix (ECM), degradation of existing ECM components and ECM protein fiber alignment. Hereby, collagen type 1 (col-I) fiber alignment, also called col-I strap formation (SF), results from the cell traction force, whereby the cell pulls the col-I fibers into bundles close around itself in order to migrate. In contrast, amoeboid migrating cells do not rely on matrix remodeling for propagation [7].

Integrins are bidirectional communicators between the intracellular cytoskeleton and the ECM proteins. Integrin-mediated signaling pathways control metastasis-related processes like cancer cell migration [8], and multiple integrins and associated cytoplasmic kinases (FAK, ERK) are upregulated by radiation in relation with a higher aggressiveness of the cancer cells [9, 10, 11, 12]. Non-muscle myosin II (NMMII) is an important regulator of cell adhesion, migration and matrix organization [13]. NMMII isoforms A and B have distinct roles in matrix organization [14], while NMMII isoform C plays an essential role in cytokinesis [15].

Previously, Blockhuys *et al.* described an increased col-I SF potential of breast cancer cells after X-radiation. They reported that integrin β1 functionality is essential for col-I SF by breast cancer cells after radiation, and that the RI increase in col-I SF potential of breast cancer cells is dependent on an increased NMMIIA expression level [16].

In this study, we evaluated the effect of X-radiation on the col-I SF potential of different colon cancer cell lines and their related behaviors. SW480 and SW620 cell lines, which originate from a primary colon adenocarcinoma and a positive lymph node obtained one year later from the same patient, respectively, facilitated the investigation of mesenchymal and amoeboid cell migration patterns, respectively [17, 18]. The two HCT116 cell lines, HCT116 p53^+/+^ (p53 wild type) and HCT116 p53^−/−^ (p53 null; p53 gene was disrupted by homologous recombination), elucidated the role of p53 in the radiation response of colon cancer cells [19]. Our study indicates that col-I SF is a potential mechanism of colon cancer cells with increased migration potential after X-radiation.

## RESULTS

### X-radiation enhanced col-I SF potential of different colon cancer cells

**Figure 1:**
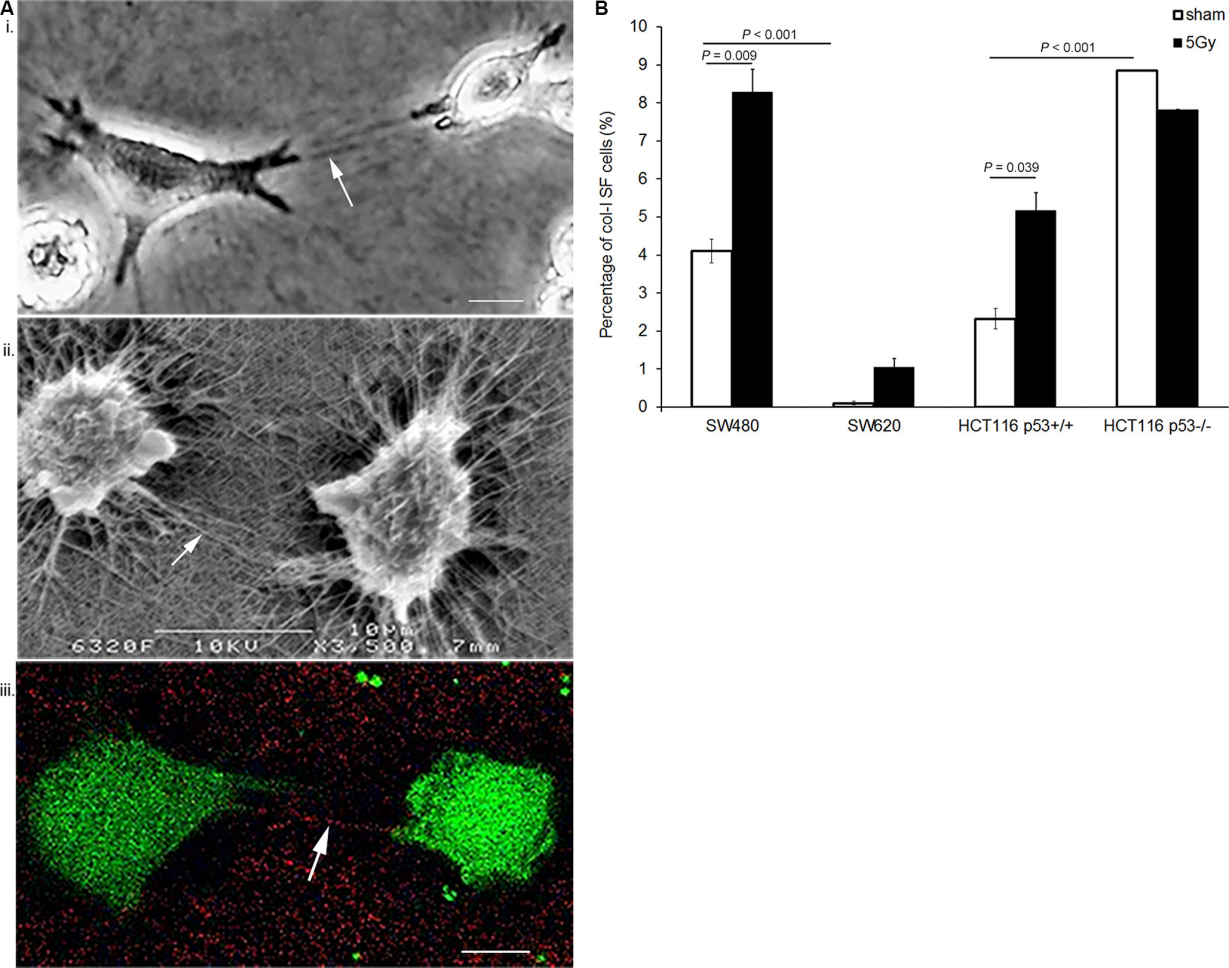
X-radiation enhanced col-I SF potential of various colon cancer cell lines (**A**) Visualization of col-I straps induced by SW480 cells in the col-I matrix assay: (i) phase-contrast microscopy (PCM), (ii) scanning electron microscopy (SEM), and (iii) second harmonic generation (SHG; red color) in combination with two-photon emission fluorescence (TPEF, green color). Visualization of the col-I straps by SHG confirmed the col-I specificity of the straps. (Arrows indicate col-I straps; scale bar = 10 μm). (**B**) Quantification of col-I SF potential of four colon cancer cell lines at day 5 after sham or 5 Gy X-radiation. Error bar represents the standard error of the mean (*n_IrExp_* = 3; *t-test*).

Cell-induced col-I straps were visualized using three microscopy techniques: phase-contrast microscopy (PCM), scanning electron microscopy (SEM), and label-free non-linear microscopy (NLM), namely, second harmonic generation (SHG) for visualization of col-I in combination with two-photon excitation fluorescence (TPEF) for cells. The images presented in Figure 1A illustrate col-I SF by SW480 cells, where col-I fibers are organized as parallel aligned col-I fibers originating from the cellular extensions with a perpendicular orientation towards the cell periphery. In addition to the two-dimensional (2D) overview of the *in vitro* system obtained by PCM and SEM, NLM acquisition resulted in a three-dimensional (3D) visualization of cell-induced col-I matrix remodeling (Supplementary Figure 1A).

Quantitative evaluation of col-I SF using PCM indicated a significantly higher col-I SF potential of SW480 vs. SW620 cells (*P* < 0.001), and a significantly lower col-I SF potential of HCT116 p53^+/+^ vs. HCT116 p53^−/−^ cells (*P* < 0.001). After 5 Gy X-radiation, col-I SF potentials of both SW480 and HCT116 p53^+/+^ cells were significantly increased (*P* = 0.009 and *P* = 0.039, respectively). Furthermore, X-radiation did not significantly change the col-I SF potentials of SW620 and HCT116 p53^−/−^ cells (Figure 1B). An X-ray dose-dependency study with SW480 and HCT116p53^+/+^ cells indicated 5 Gy as the X-ray dose with significantly increased col-I SF potentials of both cell lines (*P* < 0.001 and *P* = 0.013, respectively; Supplementary Figure 2).

Further functional implications of col-I SF by colon cancer cells were studied by the 3D col-I contraction assay, whereby col-I matrix contraction reflected the cell traction force applied to the col-I matrix. As shown in Supplementary Figure 1B, the RI increase in col-I matrix remodeling was confirmed by a trend of increased col-I matrix contraction for both HCT116 p53^+/+^ and HCT116 p53^−/−^ cells after X-radiation. No results could be presented for SW480 and SW620 cells. The experimental set up was not feasible for the SW cells, since they did not intercalate in the col-I matrix during col-I polymerization.

### RI increase in col-I SF potential related with increased motility of different colon cancer cell lines

The 15 h PCM time-series demonstrated the heterogeneity in colon cancer cell behavior and related changes in the col-I matrix. Col-I SF was observed as a dynamic process orchestrated by the cells, where col-I straps appeared and disappeared over time. Moreover, the col-I straps were generally formed in between two cells and promoted the migration of the cells towards each other. Representative videos and final PCM images for sham and X-radiated HCT116 p53^+/+^ cells are presented in Supplementary Videos 1–2 and Supplementary Figure 3A–3B, respectively. In addition to col-I SF, we observed two other phenomena: (1) col-I matrix degradation by SW480 cells (Supplementary Video 3), and (2) long distance movement of a large (leader) cell accompanied by a small (follower) cell for X-radiated HCT116 p53^+/+^ cells (Supplementary Video 4).

**Figure 2:**
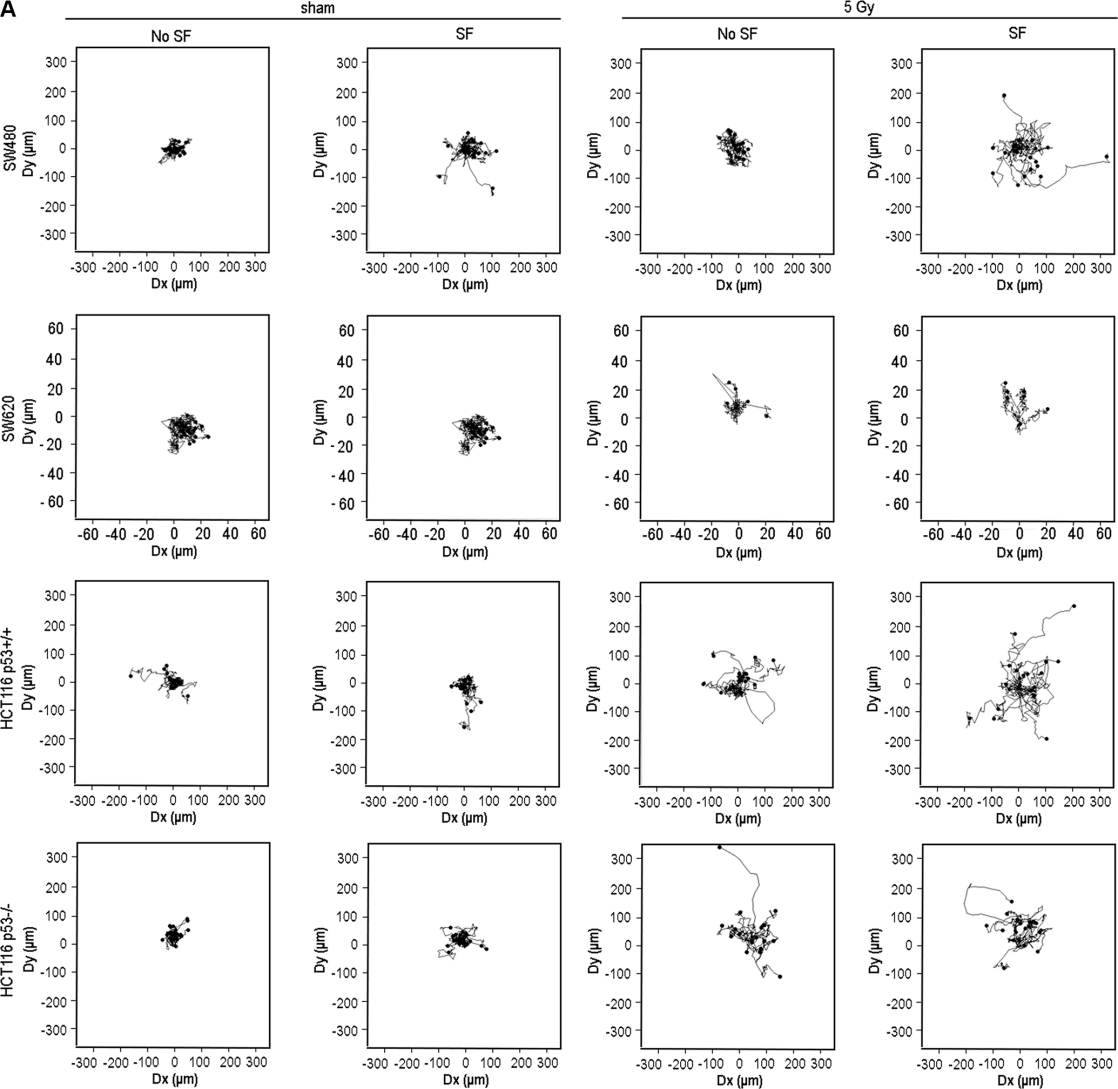

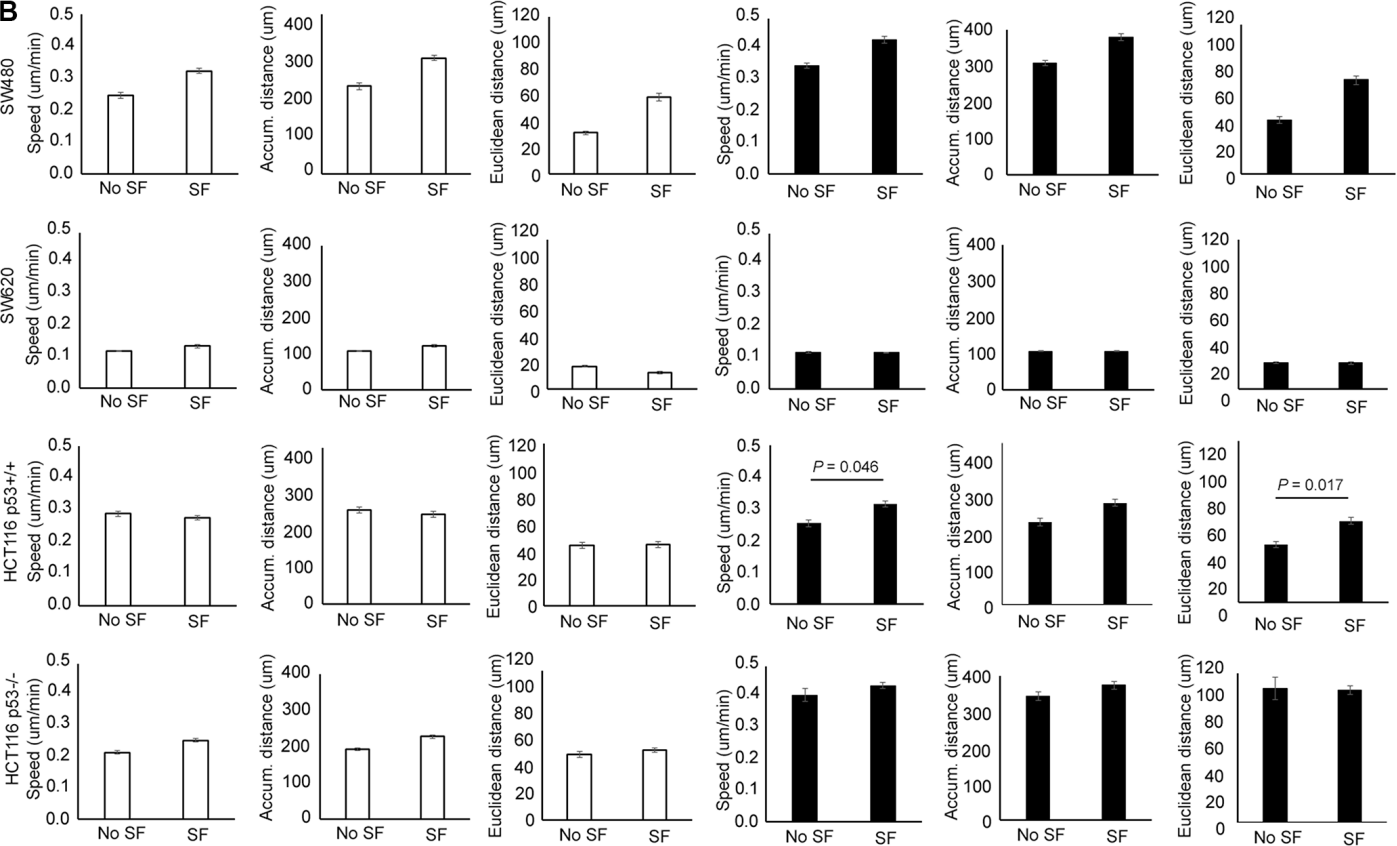
Radiation-induced increase in col-I SF potential was related with increased motility of the tested colon cancer cell lines (**A**) Wind-Rose plots presenting the tracks of randomly selected cells without (No SF) and with (SF) col-I SF potential during the 15 h time-series performed with both sham and X-radiated colon cancer cells (*n_cells_* = 20 for all conditions, except of SW620 5 Gy with *n_cells_* = 7). (B) Mean speed, accumulated distance (Accum. distance) and Euclidean distance of cells without (No SF) and with (SF) col-I SF potential during the 15 h time-series performed with both sham and X-radiated colon cancer cells. Error bar represents the standard error of the mean (*n_IrExp_* = 1 for SW480, SW620 and HCT116 p53^−/−^ cells; *n_IrExp_* = 3 for HCT116 p53^+/+^ cells; *t-test* performed for HCT116 p53^+/+^ cells only).

Single cell tracking analysis, presented as Wind-Rose plots in Figure 2A, illustrates the increase in cell motility associated with col-I SF potential for SW480 in sham or X-radiated condition and for HCT116 p53^+/+^ in X-radiated condition only, but not for HCT116 p53^−/−^ and SW620, in either sham or X-radiated condition, and HCT116 p53^+/+^ in sham condition. Further quantitative evaluation of the cell tracks supported a direct relationship between the Wind-Rose plots and the cell motility-related parameters cell speed, accumulated - and Euclidean distances. More precisely, we measured a trend of increased speed, accumulated - and Euclidean distances for SF vs. non-SF SW480 in both sham and X-radiated conditions, and a significant increase in speed (*P* = 0.046) and Euclidean distance (*P* = 0.017) for SF vs. non-SF HCT116 p53^+/+^ cells in X-radiated condition (Figure 2B).

Comparison of the motility of the four cell lines in sham condition indicated a trend of lower motility for SW620 in comparison to SW480 and HCT116 cells. After X-radiation, we observed a trend of increased speed (Supplementary Figure 4A), accumulated distance (Supplementary Figure 4B) and Euclidean distance (Supplementary Figure 4C) for SW480 and HCT116 p53^−/−^, but not for HCT116 p53^+/+^ and SW620 cells.

### X-radiation decreased adhesion but increased migration of different colon cancer cell lines

**Figure 3:**
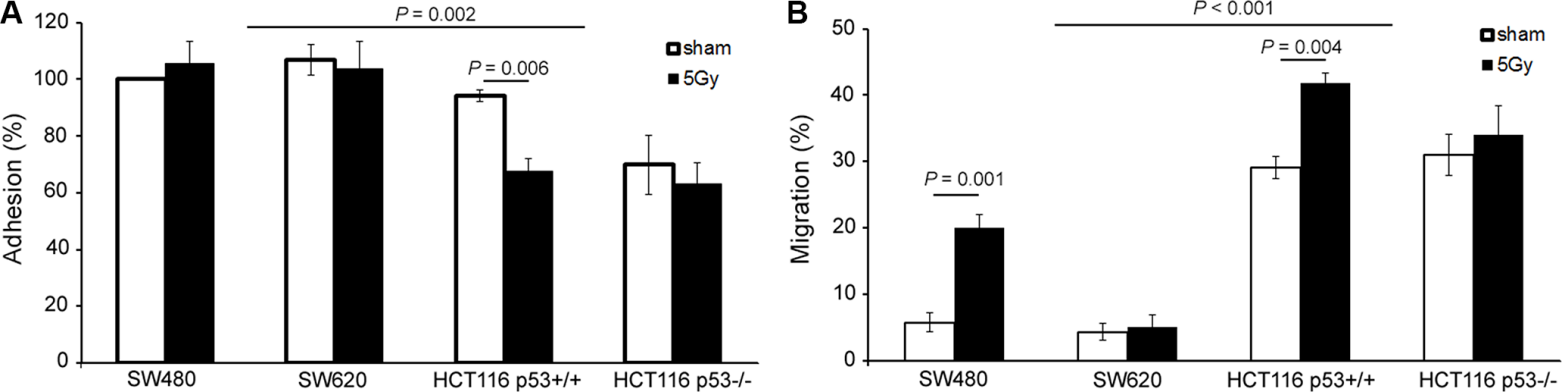
X-radiation decreased adhesion, but increased migration potential of the elucidated colon cancer cell lines (**A**) Cell adhesion potential determined by standard cell adhesion assay on col-I coating. Error bar represents the standard error of the mean (*n_IrExp_* = 3, *t-test*). (**B**) Cell migration potential determined by the zone exclusion assay on col-I coating. Error bar represents the standard error of the mean (*n_IrExp_* = 3, *t-test*).

The col-I adhesion potential was not different between SW480 and SW620 cells or between HCT116 p53^+/+^ and HCT116 p53^−/−^ cells, but a significant decrease in adhesion was observed for HCT116 vs. SW cells (*P* = 0.002). Irradiation did not change the adhesion potentials of SW480, SW620, and HCT116 p53^−/−^ cells, but resulted in a significant decrease in adhesion of HCT116 p53^+/+^ cells (*P* = 0.006) (Figure 3A).

The col-I migration potential was not different between SW480 and SW620 cells or between HCT116 p53^+/+^ and HCT116 p53^−/−^cells, but a significant increase in migration was observed for HCT116 vs. SW cells (*P* < 0.001). Irradiation did not change the migration potentials of SW620 and HCT116 p53^−/−^ cells, but resulted in a significant increase in migration of SW480 and HCT116 p53^+/+^ cells (*P* = 0.001 and *P* = 0.004, respectively) (Figure 3B).

### Integrin β1 functionality was essential for col-I adhesion and SF by colon cancer cells after X-radiation

**Figure 4:**
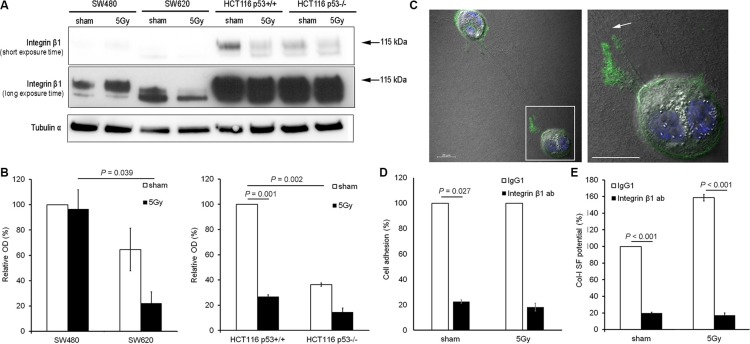
Integrin β1 functionality was essential for col-I adhesion and col-I SF by colon cancer cells after X-radiation (**A** and **B**) Representative blots (short and long exposure times) and quantitative results for integrin β1 expression in the four colon cancer cell lines for sham and 5 Gy X-radiated conditions. Hereby, the mature form of integrin β1 was quantified (arrows indicate the protein bands with molecular weight of 115 kDa). Tubulin α was used as loading control. Error bar represents the standard error of the mean (*n_IrExp_* = 3, *t-test*). (**C**) Visualization of total integrin β1 expression and col-I fiber straps formed by 5 Gy X-radiated HCT116 p53^+/+^ cells using confocal fluorescence microscopy and differential interference contrast microscopy, respectively (right panel shows framed area indicated in left panel; arrow indicates col-I SF at integrin β1-rich tip of cell protrusion; scale bar = 20 μm). (**D**) Quantification of the col-I adhesion potential of HCT116 p53^+/+^ cells in sham or 5 Gy irradiated condition, after control (IgG1) or neutralizing integrin β1 antibody (10 μg/mL) treatment. Error bar represents the standard error of the mean (*n_IrExp_* = 3). (**E**) Quantification of the col-I SF potential of HCT116 p53^+/+^ cells in sham or 5 Gy X-radiated condition after control (IgG1) or neutralizing integrin β1 antibody (10 μg/mL) treatment. Error bar represents the standard error of the mean (*n_IrExp_* = 3; *t-test*).

Integrin β1 expression analysis indicated that integrin β1 expression levels were higher in HCT116 than in SW cells. In sham condition, a trend of higher expression of the mature form of integrin β1 (MW = 115 kDa) was detected in SW480 vs. SW620 cells (100% vs. 65%, respectively), and a significantly higher expression of mature integrin β1 was determined for HCT116 p53^+/+^ vs. HCT116 p53^−/−^ cells (100% vs. 36%; *P* = 0.002). After X-radiation, a significant decrease in mature β1 integrin expression was observed for HCT116 p53^+/+^ (100% vs. 27%; *P* = 0.001), but no change for SW480, SW620, or HCT116 p53^−/−^ cells (Figure 4A and 4B).

Further investigation of the role of integrin β1 in col-I SF potential of HCT116 p53^+/+^ cells by confocal microscopy, indicated an accumulation of integrin β1 at the tip of the cell protrusions connected to the col-I strap (Figure 4C). Functional inhibition of integrin β1 using a neutralizing antibody reduced the adhesion (*P* = 0.027 in sham condition; Figure 4D) and SF potentials (*P* < 0.001; Figure 4E) of HCT116 p53^+/+^ cells in both sham and X-radiated conditions.

### X-radiation did not induced significant changes in MLC2 or NMHCIIA, -B, and –C expression

**Figure 5:**
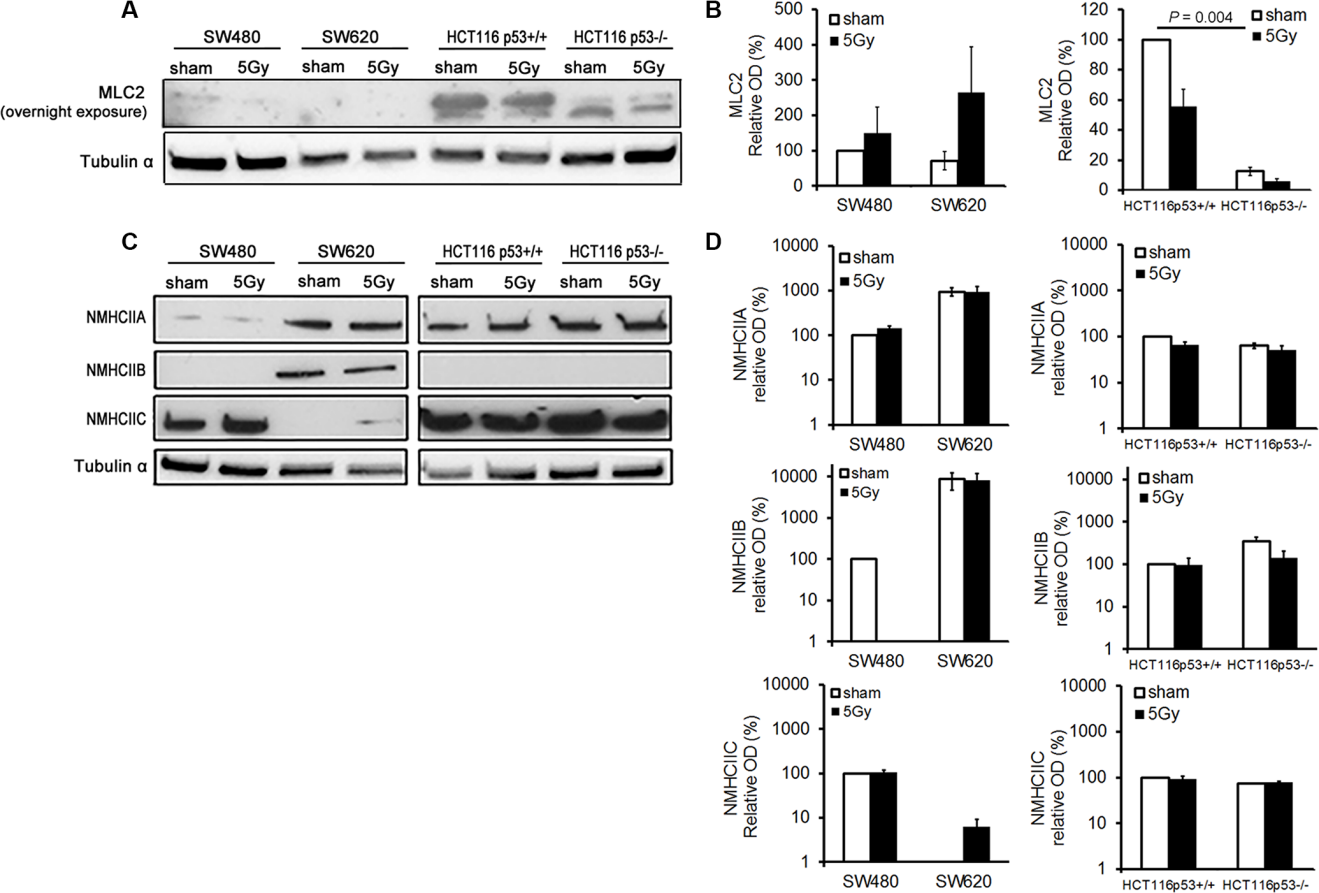
X-radiation did not induce significant changes in MLC2 and NMHCIIA, –B, and –C expression levels in the analyzed colon cancer cell lines (**A** and **B**) Representative blot (overnight exposure time) and quantitative results for MLC2 expression. Tubulin α expression was used as loading control. Error bar represents the standard error of the mean (*n_IrExp_* = 3). (**C** and **D**) Representative blot and quantitative results for NMHCIIA, –B, and –C expression levels. Tubulin α expression was used as loading control. Error bar represents the standard error of the mean (*n_IrExp_* = 3). (NMHCII, non-muscle myosin heavy chain II).

MLC2 expression was significantly higher in HCT116 p53^+/+^ than in HCT116 p53^−/−^ cells (*P* = 0.004). X-radiation resulted in a trend of increased and decreased MLC2 expressions in SW and HCT116 cells, respectively (Figure 5A and 5B). Under sham conditions, NMHCIIA was detected in all cell lines, NMHCIIB was detected in SW620 cells only, and NMHCIIC was detected in all cell lines except of SW620 cells. After X-radiation, no change in expression of the myosin II isoforms was detected in the tested cell lines, apart from a RI expression of NMHCIIC in SW620 cells (Figure 5C and 5D).

## DISCUSSION

Our study indicated col-I SF as a mechanism upregulated by colon cancer cells with increased migration potential after X-radiation. We observed higher motility and migration potentials of SW480 vs. SW620 cells, which is in line with the results described by Kubens *et al*. [18], namely, a higher spontaneous motility of SW480 vs. SW620 cells; and higher col-I SF and migration potentials of HCT116 p53^−/−^ vs. HCT116 p53^+/+^ cells, which confirms what was reported in literature [20], namely, p53 loss associated with increased cell motility. RI increase in col-I SF potential has earlier been demonstrated for breast cancer cells with the focus on cell survival but not cell migration [16]. To the best of our knowledge, the findings presented here are the first reporting the direct relationship between increased col-I SF potential and migratory behaviors of cancer cells after X-radiation.

Our study results indicate that integrin β1 is essential but not responsible for RI increase in col-I SF by colon cancer cells. In literature, multiple studies report an upregulation of integrin β1 expression associated with the aggressive phenotype and invasion after X-radiation [9, 12, 21, 22]. Besides integrin α2β1, integrin α_1_β_1_ is a mediator of col-I specific cell interactions. Li *et al.* [12] reported that integrin α_*1*_ was downregulated after radiation. Moreover, integrins α_1_β_1_ and α2β1 have been reported with contrasting roles, suggesting that decreased integrin α_1_ might also favor the increased invasiveness of irradiated cells. Our study shows the differences in NMMII isoforms A, B and C expressions between the different colon cancer cells, but indicates no change after irradiation except of a RI NMHCIIC expression in SW620 cells. NMHCIIB, which was expressed in SW620 only, is known to regulate cell polarity in non-migrating cells [23]. This observation suggests that NMHCIIB is a potential indicator of non-migrating cells in our *in vitro* model. Further, we determined a lower MLC2 expression in SW vs. HCT116 cells, and X-radiation resulted in increased and decreased MLC2 expressions in SW480 and HCT116 p53^+/+^ cells, respectively. These results for integrin β1 and NMMII-related protein expression were further supported by our PCR results for sham vs. X-radiated SW480 and HCT116 p53^+/+^ cells (see Supplementary Table 1). However, our protein expression results are not in line with the results described by Blockhuys *et al.* [16], namely, X-radiation of MCF-7/6 breast cancer cells resulted in increased NMMIIA and MLC2 expression levels in parallel with their increased col-I SF potential. Our molecular study suggest that other cellular molecules are responsible for the RI increase in col-I SF and migration potentials of colon cancer cells. Vinculin, which was recently reported by Thievessen *et al.* [24] as regulator of traction force generation by cells in a 3D collagen without affecting myosin II, is a potential candidate protein.

NLM provided 3D visual, chemically and semi-quantitative information about cell-induced col-I fiber straps. SHG and TPEF allowed 3D visualization of col-I and reactive oxygen species-positive colon cancer cells, respectively. Although SHG confirmed col-I specificity of the cell-induced fiber straps, only the thicker fibers within the straps could be visualized due to the lower resolution of our SHG set up (approximately 500 nm) (see Supplementary Figure 5). Nonetheless, our study illustrated NLM as a useful tool in biological science and clinical practice.

In the future, we will aim to evaluate the clinical significance of col-I SF in the progression and treatment outcome of rectal cancer patients. Additionally, more efforts need to be addressed to the unraveling of molecular mechanisms involved in RI aggressive behaviors of cancer cells. Hereby, our findings motivate a single cell approach using NLM. This research is crucial to identify new biomarkers and to develop novel drug targets with improvement of rectal cancer outcome.

## MATERIALS AND METHODS

### Cell lines

SW480 and SW620 human colon cancer cell lines were obtained from American Type Culture Collection (LGC standards, Teddington, UK). Both SW cell lines were maintained at 37°C and 5% CO_2_ in Eagles MEM (Sigma-Aldrich, St. Louis, MO), supplemented with 10% heat inactivated fetal bovine serum (GIBCO, Invitrogen, Paisley, UK), 0.5% L-glutamine (GIBCO), and 1% penicillin/streptomycin cocktail (GIBCO). The HCT116 p53^+/+^ and HCT116 p53^−/−^ isogenic human colon cancer cell lines were kindly provided by Professor Bert Vogelstein (Johns Hopkins University, Baltimore, MD). Both HCT116 cell lines were maintained at 37°C and 5% CO_*2*_ in McCoy's 5A medium (modified) (GIBCO), supplemented with 10% heat inactivated fetal bovine serum, 0.5% L-glutamine, and 1% penicillin/streptomycin cocktail.

### Antibodies

The following antibodies were used: mouse IgG1 (MG100) from Life Technologies (Carlsbad, CA), anti-integrin β1 P5D2 (ab24693) from Abcam (Cambridge, UK), anti-tubulin α (T9026) from Sigma-Aldrich, HRP-conjugated polyclonal goat anti-mouse or anti-rabbit antibodies from DAKO Cytomation (Glostrup, DK), and anti-integrin β1 (#4706), anti-MLC2 (#3672), anti-NMHCIIA (#3403), anti-NMHCIIB (#3404), anti-NMHCIIC (#8189), Alexa Fluor^®^ 488-conjugated anti-mouse IgG (H+L) antibodies from Cell Signaling Technologies (Danvers, MA).

### X-radiation

Cells were exposed to 5 Gy radiation dose of 6 MV photon spectrum using a linear accelerator (Clinac 4/100, Varian, Palo Alto, CA) and evaluated 5 days later. The cells were positioned below 3 cm polymethyl methacrylate, 105 cm from the photon source (source-to-surface distance (SSD) = 100 cm). The dose rate at the position of the cells was 4.8 Gy/min and the field size at SSD was 30 × 30 cm^2^. In contrast to the X-radiated cells, the sham-treated cells were not exposed to X-rays. The 5 Gy X-radiation dose used in this study is a clinical relevant dose, as it is used for short-term radiotherapy of rectal cancer patients.

### Col-I based assays

### Col-I matrix assay

A single cell suspension (10^5^ cells/1.5 ml) was seeded on top of a col-I matrix (1 mg/ml) followed by an incubation period of 24 h at 37°C and 5% CO_2_. Cell morphology and col-I remodeling were studied by an inverted PCM (AxioVert.A1, ZEISS; Software AxioVision 4.8). Col-I SF potential of the cells was calculated as the percentage of SF cells vs. the total number of adherent cells. Data was collected for three independent radiation experiments, with five randomly selected microscopic fields using a 10X objective [16].

### Stressed col-I matrix contraction assay [25]

Single cells suspended in col-I gel solution (2 × 10^5^ cells/300 μl) were added into a well of a 24-well plate. After gel polymerization, culture medium (700 μl) was added on top of the cell-containing col-I gel. After an incubation period of 24 h the col-I gel was detached from the well and contraction was measured 24 h later (contraction index (%) = (D_*well*_ − D_*col*_)/D_*well*_) × 100) (D = diameter). Data was collected for two independent radiation experiments performed in duplicate.

### Cell adhesion assay (col-I coating)

Single cell suspensions (10^5^ cells/500 μL) were seeded in col-I pre-coated wells of a 24-well plate. After an incubation period of 90 min at 37°C and 5% CO_*2*_, a standard crystal violet staining was performed and the number of adherent cells was quantified after PCM using ImageJ software. Data was collected for three independent radiation experiments performed in duplicate.

### Zone exclusion assay (col-I coating)

Single cells suspended in 1% FBS (1.5 **×** 10^5^ cells/150 μL for SW480, HCT116 p53^+/+^ and HCT116 p53^−/−^, and 5 × 10^5^ cells/150 μL for SW620) were seeded in col-I pre-coated wells of the zone-exclusion assay (Oris™ migration assay-Collagen I coated, Amsbio). Cell stoppers were removed after an incubation period of 8 h at 37°C and 5% CO_*2*_. Wound closure was evaluated after another 24 h of incubation using PCM (BX41, Olympus). The migration index (%) was calculated as (D_*control*_ − D_*test*_)/D_*control*_) × 100 (D = diameter). Data was collected for three independent radiation experiments performed in duplicate.

### Integrin β1 neutralization

Single cell suspensions were pretreated for 1 h under motion at 37°C in serum-free culture medium supplemented with 10 μg/mL mouse IgG1 (isotype control) or 10 μg/mL integrin β1 neutralizing antibody.

### Western blot

Proteins were extracted by lysis buffer containing 150 mM NaCl, 2% Triton, 0.1% SDS, 50 mM Tris pH 8.0, protease inhibitor cocktail (ROCHE, Basel, CH) and phospatase inhibitor cocktail (ROCHE) and stored at −20°C. Protein concentration was determined by the colorimetric BCA protein assay reagent (Pierce, Woburn, MA). Equal amounts of protein for each sample were subjected to SDS/PAGE and transferred onto nitrocellulose membranes (Whatman, Boston, MA). Membranes were first probed with primary antibodies and subsequently with HRP-conjugated secondary antibodies. Protein bands were detected using ECL plus Western Blotting Detection System (GE Healthcare, Piscataway, NJ).

### Immunofluorescence staining

Cells seeded on top of a col-I gel were fixed and prepared for immunofluorescence staining as described by Abe *et al.* [26]. Shortly, samples were fixed with 4% paraformaldehyde (PFA), blocked with 1% glycine/2% BSA/PBS and permeabilized in 0.5% Triton-X-100/phosphate buffered saline (PBS). Samples were then incubated with primary antibody diluted in 1% BSA/PBS for 1.5 h. After washing, samples were incubated in secondary antibody diluted in 1% BSA/PBS for 1 h. Samples were washed, stained with DAPI, mounted and analyzed with confocal microscopy.

### Microscopy

### Confocal microscopy

Confocal images were captured with a Zeiss Axio Imager.Z2 equipped with a LSM700 confocal module (ZEN software) using a 63X/1.4NA oil immersion objective.

### Scanning electron microscopy (SEM)

SEM was performed using a JEOL JSM-6320F operated at 10 kV. Collagen gels plus cells were fixed with 4% PFA in PBS for 1 h at room temperature. The fixed samples were dehydrated in a graded distilled water/ethanol series: 30%, 50%, 70%, and 100% for 15 min each, followed by washes with a graded ethanol/hexamethyldisilazane series: 30%, 50%, 70%, and 100% for 15 min each, and finally allowed to dry overnight. This drying procedure has been employed to avoid sample shrinkage [27]. The collagen matrix was sputter-coated with approximately 14 nm platinum prior to SEM. SEM images were taken at multiple locations across the sample and used to qualitatively assess the col-I fiber alignment induced by the cells.

### Non-linear microscopy (NLM) [28]

A PFA-fixed sample was mounted with fluorescence mounting medium (DAKO) on a microscope slide covered with a 0.17 mm coverslip. Two laser beams of synchronized pico-second pulse trains in the near-infrared wavelength regimes were spatially and temporally overlapped and coupled into an inverted microscope. Simultaneous illumination of the sample with laser beams at wavelengths of 817 nm and 1064 nm induced the SHG and TPEF processes in our sample with combined imaging of col-I and cells, respectively. The laser beams were focused on the samples using a 40x objective (Nikon Plan Fluor N.A. 1.3, working distance 0.21 mm), and emitted SHG and TPEF signals were registered by single-photon-counting photomultiplier tubes in transmission and reflection mode, respectively. Bandpass filters 405/10 and 514/30 were used to isolate the SHG and TPEF signals, respectively. Typical average excitation powers at the samples were 5 mW and 6 mW for laser beams 817 nm and 1064 nm, respectively. Simultaneous SHG and TPEF microscopy z-stacks covered an area of 127.9 × 127.9 μm^2^ and a depth of 25 μm (step size = 0.2 μm). The image acquisition time at each position was 12.4 sec. Merged SHG and TPEF images and volume views showing the cells on col-I matrix were processed using the ImageJ “Volume Viewer” plugin.

### Phase-contrast video-microscopy

Cells were seeded on the col-I matrices at a density of 1 × 10^5^ cells/well in 2.5 mL of standard growth medium, and time-lapses were started after an incubation period of 30 min. Phase-contrast images were acquired using a LSM700 confocal mounted on a Zeiss Observer Z1 (Jena, Germany), controlled by Zeiss Zen (2012) software. Multi-position, time-lapse images were captured with 120 sec interval for 15 h at 37°C and 5% CO_*2*_. Images were captured with a phase-contrast 10X/0.25 NA objective. For the analysis, every cell in each movie that remained within the field-of-view during the entire time-series was tracked using the Manual tracking tool (point-click mode) provided by ImageJ software. In each case, the cell centroid, defined as a half point along the long distance of the cell, was used for tracking. Data were exported into Ibidi's “Chemotaxis and Migration” tool for the Wind-Rose plot representation of the tracks and the determination of the parameters speed (= accumulated distance/time), accumulated - and Euclidean distances.

### Statistical analysis

Statistical analysis of paired observations was conducted using the Student's *t-test*, whereby the threshold level of significance was set to 0.05.

## SUPPLEMENTARY MATERIALS FIGURES AND TABLES










