# Supplementary material for: X-radiation enhances the collagen type I strap formation and migration potentials of colon cancer cells
Source: Oncotarget. 2016 Sep 19;7(44):71390–9. doi: 10.18632/oncotarget.12111 (PMC5342086; doi:10.18632/oncotarget.12111)
Supplement: Supplementary file 1 [file oncotarget-07-71390-s001.pdf]

X-radiation enhances the collagen type I strap formation and migration potentials of colon cancer cells

Supplementary Materials

SUPPLEMENTAL MATERIAL FOR SUPPLEMENTARY TABLE 1

RNA extraction and cDNA preparation

Total RNA was extracted from SW480 and HCT116 $p53^{+/+}$ cells at 24 h after sham or 5Gy X-radiation (n_{IrExp} = 3) using the RNeasy Mini Kit (Qiagen, Hilden, Germany) according to the manufacturer's instructions. Quality of RNA samples was evaluated using RNA 6000 Nano Kit (Agilent Technologies, Santa Clara, CA). A total of 1 μg mRNA was reverse-transcribed to cDNA with RT^2 first strand kit (Qiagen). Each cDNA sample was prepared for qRT-PCR.

PCR array and data analysis

The RT^2 Profiler PCR Array system for human cell motility (Qiagen) was used according to the manufacturer's protocol. Briefly, 102 μL cDNA was mixed with 2× RT^2 SYBR Green Master Mix (Qiagen) and H_2O to a total volume of 2700 μL. Subsequently, 25 μL of the mixture was placed into each well of the 96-well PCR array plate. The PCR program of one cycle of 10 min at 95°C, followed by 40 cycles of 15 sec at 95°C and 1 min at 60°C, was performed on the ABI-7300 instrument (Applied Biosystems, Waltham, MA). The threshold cycle changes (ΔCt) denoted the difference in Ct for the gene of interest based on the Ct level of GAPDH within the sample. The relative expression intensity was obtained by calculating the $2^{-\Delta Ct}$ for each cell line in both sham and X-radiated conditions for further *t*-test analysis, according to RT^2 Profiler PCR Array Data Analysis v3.5 Handbook (Qiagen).

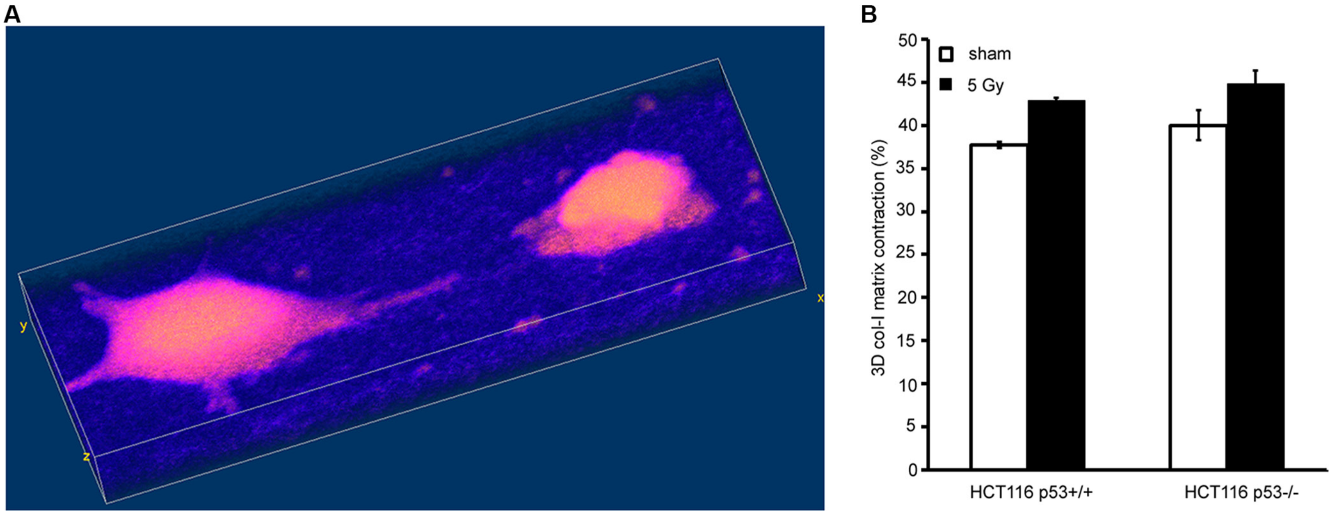

Supplementary Figure S1: (**A**) Label-free 3D visualization of col-I straps by 5 Gy X-radiated SW480 cells using NLM, whereby col-I and cells are visualized using SHG and TPEF, respectively. (**B**) Quantification of 3D col-I matrix contraction by HCT116 $p53^{+/+}$ and HCT116 $p53^{-/-}$ cells at day 5 after sham or 5 Gy X-radiation. Error bar represents the standard error of the mean (n_{IrExp} = 2).

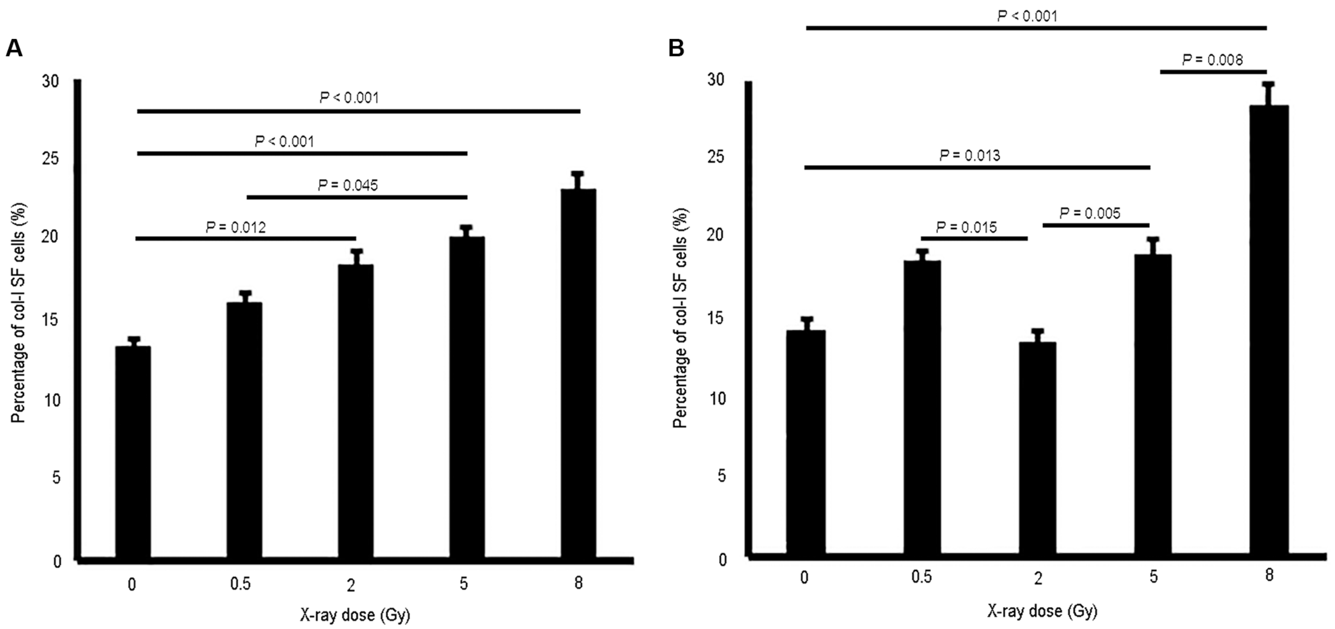

Supplementary Figure S2: Col-I SF potential of SW480 and HCT116 p53$^{+/+}$ cells at day 5 after irradiation with 0, 0.5, 2, 5 and 8 Gy X-radiation. Error bar represents the standard error of the mean (n_{IrExp} = 3; *t*-test).

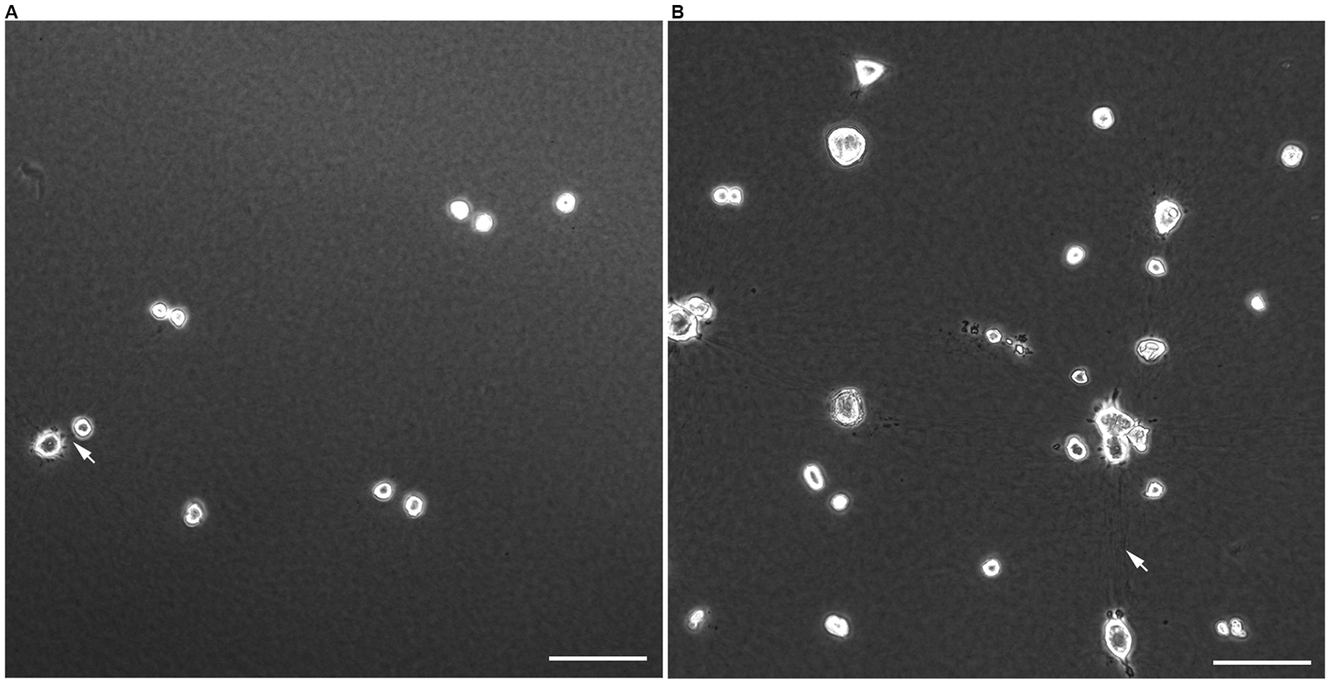

Supplementary Figure S3: PCM images obtained at the end of the time series with (A) sham- and (B) 5 Gy X-radiated HCT116 p53$^{+/+}$ cells, corresponding with Supplemental Videos 1 and 2, respectively. (Arrows indicate col-I fiber straps; scale bars = 50 μm).

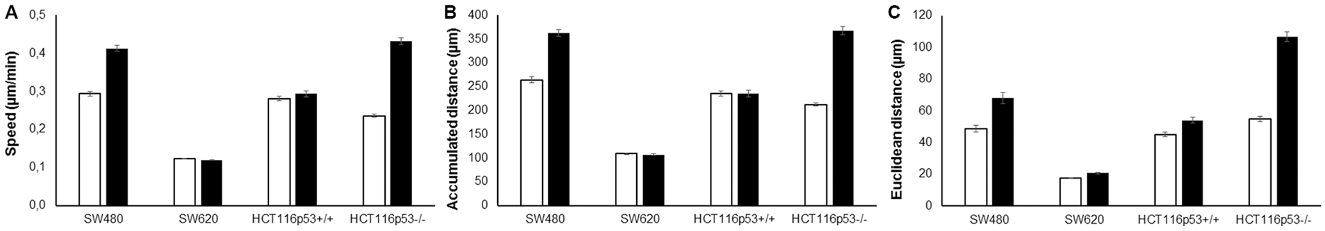

Supplementary Figure S4: Impact of col-I SF potential on the behavior of colon cancer cells in sham or X-radiated condition. (**A**) Mean speed of sham or 5 Gy X-radiated cells on a col-I matrix. Error bar represent the standard error of the mean (n_{IrExp} = 1 for SW480, SW620 and HCT116 p53$^{-/-}$; n_{IrExp} = 3 for HCT116 p53$^{+/+}$). (**B**) Mean accumulated distance of sham or 5 Gy X-radiated cells on a col-I matrix. Error bar represents the standard error of the mean (n_{IrExp} = 1 for SW480, SW620 and HCT116 p53$^{-/-}$; n_{IrExp} = 3 for HCT116 p53$^{+/+}$). (**C**) Mean Euclidean distance of sham or 5 Gy X-radiated cells on a col-I matrix. Error bar represents the standard error of the mean (n_{IrExp} = 1 for SW480, SW620 and HCT116 p53$^{-/-}$; n_{IrExp} = 3 for HCT116 p53$^{+/+}$).

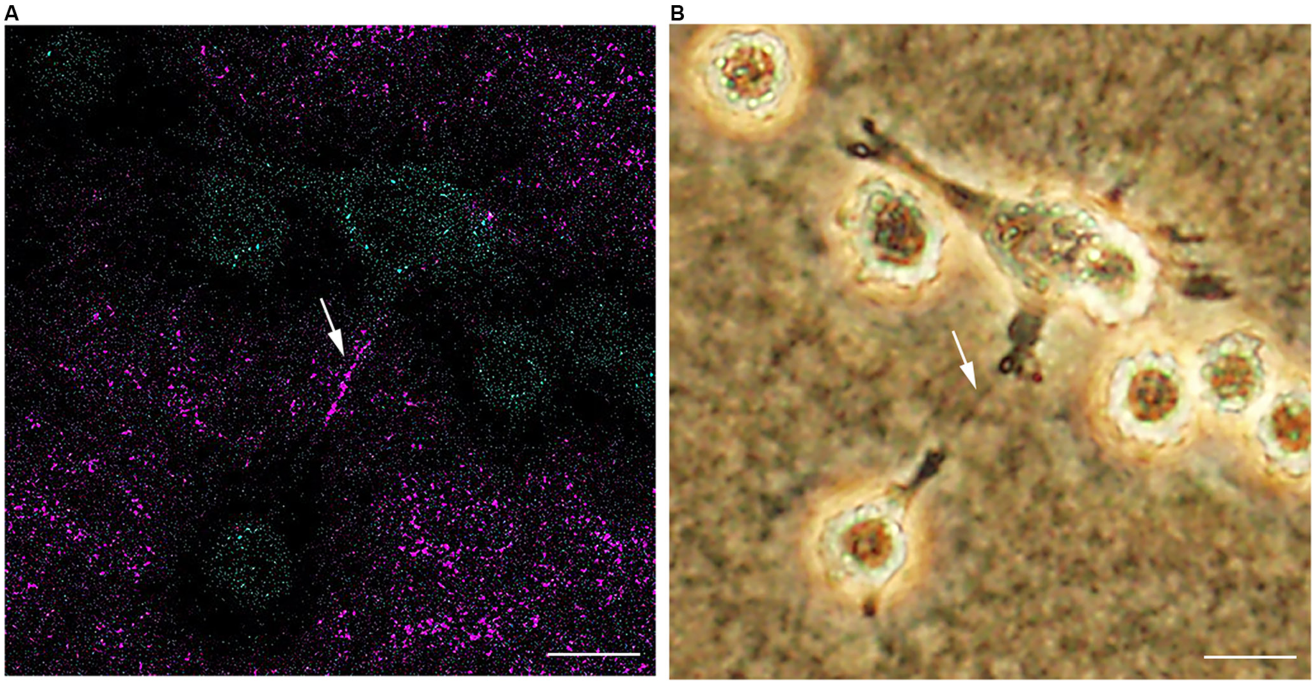

Supplementary Figure S5: NLM (SHG+TPEF) (A) and PCM (B) images of 5 Gy X-radiated SW480 cells on a col-I matrix. (Arrows indicate the col-I fiber visualized by both SHG and PCM; scale bars = 20 µm).

Supplementary Table S1: RNA expression level of integrin and myosin II-related genes determined by the RT^2 Profiler™ Cell motility PCR array performed with RNA extracted from SW480 and HCT116 p53$^{+/+}$ cells at 24 h after sham or 5 Gy X-radiation

Gene symbol	Gene name	SW480		HCT116 p53$^{+/+}$	
		Fold change	*P*-value	Fold change	*P*-value
ITGB1	Integrin beta 1	−1.40	0.097	−1.06	0.630
ILK	Integrin-linked kinase	−1.60	0.007	−1.29	0.004
PTK2	Focal kinase 1	−1.25	0.195	−1.03	0.788
PTK2B	Focal kinase 2	−1.63	0.005	1.25	0.211
MYH9	Non-muscle myosin heavy chain II-A	−1.40	0.236	−1.12	0.613
MYH10	Non-muscle myosin heavy chain II-B	−1.19	0.301	−1.08	0.810
MYL9	Myosin light chain II	−1.21	0.354	1.26	0.478
MYLK	Myosin light chain kinase	−1.13	0.501	1.17	0.010

Supplementary Video S1: 15 h time-series from sham treated HCT116 p53$^{+/+}$ cells on col-I matrix.

Supplementary Video S2: 15 h time-series from 5 Gy X-radiated HCT116 p53$^{+/+}$ cells on col-I matrix.

Supplementary Video S3: 4 h time-series from 5 Gy X-radiated SW480 cells on col-I matrix.

Supplementary Video S4: 6 h time-series from 5 Gy X-radiated HCT116 p53$^{+/+}$ cells on col-I matrix.
